# Interference With Coagulation Cascade as a Novel Approach to Counteract Cisplatin-Induced Acute Tubular Necrosis; an Experimental Study in Rats

**DOI:** 10.3389/fphar.2018.01155

**Published:** 2018-10-11

**Authors:** Mohamed G. Ewees, Basim A. S. Messiha, Ali A. Abo-Saif, Asmaa M. A. Bayoumi, Mohamed S. Abdel-Bakky

**Affiliations:** ^1^Department of Pharmacology and Toxicology, Faculty of Pharmacy, Nahda University, Beni-Suef, Egypt; ^2^Department of Pharmacology and Toxicology, Faculty of Pharmacy, Beni Suef University, Beni-Suef, Egypt; ^3^Department of Pharmacology, Faculty of Medicine, Al-Azhar University, Cairo, Egypt; ^4^Department of Biochemistry, Faculty of Pharmacy, Minia University, Minia, Egypt; ^5^Department of Pharmacology and Toxicology, Faculty of Pharmacy, Al-Azhar University, Cairo, Egypt

**Keywords:** coagulation cascade, cisplatin, rivaroxaban, fibrin, tissue factor, nephrotoxicity

## Abstract

Coagulation system activation plays an important role in the pathophysiology of different diseases. In spite of massive research regarding cisplatin-induced nephrotoxicity, the role of coagulation cascade in such toxicity is still questionable. Here, we aim to investigate the role of activation of coagulation system in the initiation of cisplatin-induced acute renal tubular necrosis. Moreover, the role of the anticoagulant rivaroxaban against such toxicity was investigated. Briefly, animals were classified into seven groups, eight rats each. Group 1 served as normal control group, groups (2–7) received i.p. single doses of cisplatin (6 mg/kg b.w), groups (6–7) were treated with rivaroxaban (5 and 7 mg/kg b.w, p.o., respectively) 7 days before cisplatin injection and completed for 4 days. Animals in groups (2, 3, and 4) were sacrificed after 1, 2 and 3 days of cisplatin injection, respectively, while groups (1, 5, 6, and 7) were sacrificed after 4 days of cisplatin injection. Serum cystatin-c, urea, creatinine and γ-glutamyl transferase, urinary Lipocaline-2, and KIM-1 protein densities, as well as glomerular filtration rate (GFR) were assessed. Immunofluorescence examination of glomeruli fibrin and tissue factor (TF) was also performed coupled with a histopathological study. Cisplatin administration increased expression of fibrin and TF starting 24 h of cisplatin injection even before renal failure markers elevated. Leukocytosis, thrombocytopenia, and increased prothrombin time were also observed. Cisplatin also induced tubular damage evidenced by increased serum cystatin-c, urea, and creatinine with significant decrease in GFR and Gamma glutamyl transferase (GGT) activity. Rivaroxaban significantly decreased elevation of fibrin and TF with significant reduction in serum creatinine, BUN and cystatin-c levels. Rivaroxaban also significantly improved hematological markers and histological features as well. This study showed that activation of coagulation system plays an important role in the pathophysiology of cisplatin-induced acute renal tubular damage. Interference with coagulation cascade may be a promising nephroprotective strategy against chemical nephrotoxicity.

## Introduction

Acute tubular necrosis (ATN) is one of acute renal failure (ARF) leading cause where it represents about 85% of all ARF cases (Mehta et al., 2004). It is characterized by destruction of tubular epithelial cells with acute suppression of renal function (Tillyard et al., 2005). There has been an increase in reported cases of ARF in the last 20 years which mean that this incidence appears to be rising over time at an alarming rate (Siew and Davenport, 2015).

Acute Tubular necrosis may results from oxygen deficiency, septic or toxic kidney injury which caused by radio-contrast agents or antibacterial, antimycotic or cytotoxic drugs (Waikar et al., 2008). However, drug induced kidney injury is recognized as a major factor in about 20% of cases and its incidence may be as high as 66% among older adults (Naughton, 2008; Barrantes and Horwedel, 2012). One of these drugs which play an important role in cancer chemotherapy is cisplatin (CP).

Despite the efficacy of CP against different types of tumors, its use is limited due to sever nephrotoxicity induced after starting treatment (Saleh et al., 2014; Bhat et al., 2015). There are several theories that contributed significantly to understand the pathophysiology of CP-induced ATN including; ATP depletion, increased intracellular free Ca^2+^ concentration, mitochondrial injury with cytochrome C leakage, alteration in tubular cell structure, apoptosis, hypoxia, inflammation, reactive oxygen species (ROS) (Himmelfarb et al., 2004; Molitoris and Sutton, 2004; Lorz et al., 2006; Nangaku and Eckardt, 2007; Akcay et al., 2009).

In spite of the significance progress created in understanding the biology and mechanisms of ARF in animal models, interpretation of these findings into improved management for patients is not sufficient (Soni et al., 2009; Gupta et al., 2016), Therefore, new strategies and molecular approaches to minimize drug-induced renal toxicity have been developed. One of these new strategies need to be investigated in the pathophysiology of ARF is hyper activation of coagulation system.

Blood coagulation is one of the most important pathways in the body which has an important physiological role in control of blood hemostasis (Chu, 2005). Blood coagulation is occurred through a series of important biochemical reactions pathways including blood platelets, coagulation factors and thrombin; where they all called coagulation cascade (Guy et al., 2007; Stoll et al., 2008).

Tissue factor (TF) plays a crucial role in triggering coagulation cascade where it is induced only after endothelial wall injury (Hoffman et al., 2004; Chu, 2005). TF becomes free to bind plasma factor VII leading to activation of Factor (x), initiating the clotting cascade generation leading to fibrin production (Butenas et al., 2007; Stoll et al., 2008) which form a strong plaque to prevent bleeding. However, excessive or uncontrolled clot production leads to thrombosis blocking blood flow that supplies cells with oxygen and nutrients which lead to cell death manifests (Chu, 2005).

The pathological role of coagulation activation has also been recognized in different diseases as atherosclerosis (Koh et al., 2004), stroke (Golledge et al., 2007), cardiovascular diseases, acute and chronic inflammatory disease, fibrosis, and cancer (Bach, 2006; Chambers and Scotton, 2012). Therefore, it is very interesting to investigate the role of activation of coagulation system as a new hypothesis for elucidating the pathophysiology of CP-induced ARF.

To fulfill this purpose, a time course study was performed, fibrin and TF as markers of coagulation system activation were detected, as well as biochemical and molecular markers of renal toxicity were measured. In addition, we evaluated the possible protective effect of rivaroxaban, FXa inhibitor, to counteract CP-induced ATN through interference with coagulation cascade activation.

## Materials and Methods

### Animals

Adult male albino rats, weighing 220 ± 20 g were obtained from Nahda University Animal Facility, Beni-Suef, Egypt. The rats were kept under standard conditions of temperature (25°C ± 0.5) and relative humidity (55 ± 1) with 12-h light/dark cycles for 1 week before being subjected to laboratory experiments. Animals were fed standard diet pellets (El-Nasr Company, Abou-Zaabal, Cairo, Egypt), and allowed free access to water *ad libitum*. All animal housing and handling were conducted in compliance with the Beni-Suef University guidelines and in accordance with the research protocols established by the Animal Care Committee of the National Research Center (Cairo, Egypt) which followed the recommendations of the National Institutes of Health Guide for Care and Use of Laboratory Animals (Publication No. 85–23, revised 1985).

### Drugs and Chemicals

Rivaroxaban was purchased from LIPITIS Egypt for Pharmaceuticals and Medical Products (Cairo, Egypt). CP was obtained from Mylan Institutional LLC, (Rockford, IL, United States). Triton X-100 and paraformaldehyde were purchased from Sigma-Aldrich Co. (United States). Bovine serum albumin (BSA) was obtained from BIOMARK laboratories (India), horse serum was obtained from Sigma-Aldrich Co. (St. Louis, MI, United States), Dako solution was purchased from Dako (Carpinteria, CA, United States). DAPI (4, 6-diamidino-2-phenylindole), Fluoromount, were obtained from Sigma-Aldrich Co. (United States). All other chemicals and solvents used were of analytical grade.

### Kits and Antibodies

Creatinine and blood urea nitrogen (BUN) diagnostic kits were purchased from Diamond Laboratory Reagents (Cairo, Egypt). Rat cystatin-c ELISA kit was obtained from CUSABIO (MD, United States). Gamma glutamyl transferase (GGT) activity kinetic assay kit was purchased from HUMAN Biochemical and Diagnostic (Germany). Prothrombin time (Pt) and partial thromboplastin time (Ptt) kits were obtained from LABiTec GmbH (Ahrens Burg, Germany). Rat Lipocalin-2/NGAL and KIM-1 polyclonal antibodies were obtained from R&D Systems (MO, United States). Anti-TF mouse monoclonal antibody was purchased from Thermo Scientific Pierce (IL, United States). Goat anti-mouse Alexa fluor 488 was obtained from Invitrogen (TX, United States). Polyclonal rabbit fibrin antibody was obtained from Dako (CA, United States). Cy3-conjugated Goat anti-rabbit antibody was purchased from Jackson Immunoresearch (PA, United States).

### Experimental Design

In this study, 56 male albino rats were divided into seven groups, eight rats in each. Group 1: served as control group and received saline only. Group 2; animals were treated with CP (single i.p. dose of 6 mg/kg b.w) and were sacrificed 1 day after CP injection. Group 3; animals were treated with CP (single i.p. dose of 6 mg/kg b.w) and were sacrificed 2 days after CP injection. Group 4; animals were treated with CP (single i.p. dose of 6 mg/kg b.w) and were sacrificed 3 days after CP injection. Group 5; animals were treated with CP (single i.p. dose of 6 mg/kg b.w) and were sacrificed 4 days after CP injection. Groups (6 and 7); animals were treated orally with rivaroxaban (5 and 7 mg/kg b.w), respectively (see **Supplementary Data**), 7 days before CP injection and 4 days after injection then were sacrificed. The dose of CP was selected to induce nephrotoxicity in accordance with the method reported by Hosseinian et al. (2016).

Blood samples were used for determination of serum creatinine (Sr. Cr), BUN, cystatin-c, complete blood count (CBC), Pt and Ptt. Animals were housed separately on metabolic cages 24 h before end of the experiment for urine samples collection. Urine samples were used for determination of glomerular filtration rate (GFR), GGT activity, urinary kidney injury molecule-1 (KIM-1) and urinary lipocaline-2 levels. Tissue samples were fixed in Davidson’s solution and used for immunofluorescence detection of fibrin and TF and histological examination.

### Biochemical and Molecular Detection of Kidney Functions Tests

Serum creatinine, BUN, and urine creatinine concentration were determined according to the manufacturer’s instructions (Richard et al., 1974; Charles and Crouch, 1977), and GFR were calculated using standard formulae (Quiros et al., 2010). Serum cystatin-c level was determined using ELISA kits, however; GGT enzymatic activity was measured by kinetic assay kit according to Lee et al. (2003).

### Hematological Analysis

#### Determination of Complete Cell Count (CBC)

Hematological parameters including total WBC, lymphocyte percent (Lymp%), mononuclear cell percent (Mon%), granulocyte percent (Gr%), RBC number count, haemoglobin (Hgb), haematocrit (Hct), mean corpuscular volume (MCV), mean corpuscular haemoglobin concentration (MCHC), red cell distribution width (RDW), platelet count (Plt), mean platelet volume (MPV) and platelet distribution width (PDW) were performed using an automated hematology analyzer, ABX Micros 60 Analyzer ( Montpellier, France).

#### Determination of Prothrombin Time (Pt) and Partial Thromboplastin Time (Ptt)

Prothrombin time and Ptt were measured according to the method described by Goyal et al. (2015) using a CoaDATA automated analyzer (Ahrensburg, Germany) according to the manufacturer’s instructions.

### Western Blotting

For estimation of the urine content of lipocalin-2 or KIM-1, 20 μl of each urine sample was subjected to SDS–PAGE (10% polyacrylamide) under reducing conditions. The SDS-sample buffer contains 62.5 mM Tris–HCl (pH 6.8), 2% SDS, 0.02% BPB, 10% glycerol and 5% β-mercaptoethanol. After the run, protein bands were transferred to a nitrocellulose membrane using a semi-dry blotter (Bio-Rad) in presence of a blotting buffer containing 100 mM Tris, 192 mM glycine, and 10% methanol. The blot was subsequently blocked with TBS-T buffer (25 mM Tris–HCl pH 7.5, 150 mM NaCl, 0.05% Tween-20), and 5% skim milk for 1 h at room temperature. The blot was incubated overnight at 4°C with the primary antibody (goat anti-lipocalin-2 or goat anti-KIM-1), followed by washing with TBS-T. Subsequently, incubation with HRP-conjugated anti-goat secondary antibody was performed for 1 h at room temperature, followed by washing with TBS-T and detecting the bands using DAB colorimetric detection kit (Chongqing Biospes Co., Cat. # BWR1069). Protein bands on the blots were quantified using Image J program, and then statistical analysis was performed using Prism 5 program.

### Immunofluorescence

The immunofluorescence staining was performed as previously described (Abdel-Bakky et al., 2011). Briefly, sections were de-paraffinized in xylene and rehydrated in graded ethanol concentrations. Antigen was retrieved using Dako solution (0.01 Msodium citrate buffer, pH6) in a microwave oven at 500 Watt for 20 min. After cooling down, sections were washed by PBST (0.05% of tween 20 in phosphate buffer saline (PBS) pH 7.4) for 9 min. After fixing with *p*-formaldehyde (3.7% for 10 min), tissues were blocked with blocking buffer (PBS containing 1% BSA and 10% horse serum) at room temperature for 1 h. Incubation with the primary antibodies (Anti-TF mouse monoclonal antibody and polyclonal rabbit fibrin antibody) in a concentration of 1:300 overnight at (4°C) was done. After washing, tissue antigens were detected by goat anti-mouse Alexa fluor 488 or goat anti-rabbit Cy3 secondary antibodies for 30 min. Counterstain was done using 4′,6′-diamidino-2-phenylindole (DAPI) and washed by PBST for 30 min. Finally, tissue sections were mounted using Fluoromount G and visualized by fluorescence microscopy Leica DM5000 B (Leica, Germany). The average fluorescence intensity of 3–5 microscopic fields was measured for each tissue section using Image-J/ NIH software and normalized to DAPI intensity.

### Histopathology

Paraffin blocks were made and tissue sections (5 μm) were cut and stained with Hematoxylin–Eosin (H&E) for histological examination using light microscope attached to a digital camera. Kidney histology was examined in a blinded fashion. The stained sections of kidney were examined for cell lysis, loss of brush border, cast formation and other nephrotoxic damages.

### Statistical Analysis

All data were expressed as mean ± standard error (S.E.) of eight rats per experimental group. Statistical analysis was performed using one-way ANOVA test followed by Tukey’s multiple comparisons test, using GraphPad Prism5 computer software (San Diego, CA, United States), with values of *p* < 0.05 considered statistically significant.

## Results

### Effect of Cisplatin (CP) With or Without Rivaroxaban (Riva) on Renal Function Tests

**FIGURE 1:**
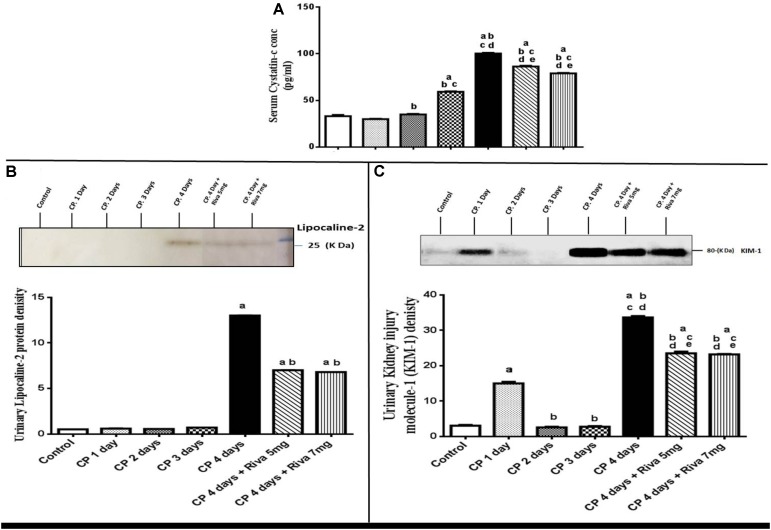
Effect of single i.p. dose of cisplatin (CP) 6 mg/kg b.w after 1, 2, 3 and 4 days with or without rivaroxaban 5 mg /kg b.w and 7 mg/kg b.w on renal function tests including; serum cystatin-c **(A)**, urinary lipocaline-2 **(B)** and urinary KIM-1 **(C)**. Data are expressed as mean ± S.E. Multiple comparisons were done using one way ANOVA followed by Tukey-Kramer as a post-ANOVA test (a) Significantly different from control group at *p* < 0.05. (b) Significantly different from CP 1 day group at *p* < 0.05. (c) Significantly different from CP 2 day group at *p* < 0.05. (d) Significantly different from CP 3 day group at *p* < 0.05. (e) Significantly different from CP 4 day group at *p* < 0.05.

Rats treated with single i.p. dose of cisplatin (6 mg/kg b.w) showed no significant change in Sr. Cr, BUN levels (**Table 1**) and cystatin-c (**Figure 1A**) after the 1st and 2nd days of CP injection as compared to normal control. Meanwhile, CP significantly increased levels of Sr. Cr, cystatin-c and BUN after 3 and 4 days as compared to normal control group (**Table 1** and **Figure 1A**). In addition, a gradual significant reduction in GGT activity was observed starting from day 1 and reached to maximum decline after 4 days of CP injection (**Table 1**). No significant changes were observed in urea/creat ratio and the level of urinary lipocaline-2 until day 3 of CP injection while these markers were significantly increased after 4 days of CP administration as compared to normal control group (**Table 1** and **Figure 1B**).

**Table 1:** Effect of cisplatin (CP) with or without rivaroxaban (Riva) on renal function tests.

Groups	Sr. Cr. (mg/dl)	BUN (mg/dl)	Urea/Creat ratio	GFR (ml/min)	GGT activity (U/L)
Normal control	0.555 ± 0.03	36.104 ± 1.03	66.52 ± 4.52	0.76 ± 0.065	337.57 ± 17.86
CP 1 day	0.54 ± 0.02	45.94 ± 2.48	85.66 ± 4.67#	1.65 ± 0.16^a^	198.54 ± 16.95^a^
CP 2 days	0.8 ± 0.06	55.95 ± 6.06	69.59 ± 4.33#	0.53 ± 0.14^b^	139.78 ± 16.46^a^
CP 3 days	1.49 ± 0.10^abc^	112.52 ± 8.89^abc^	57.44 ± 5.73^b^	0.2 ± 0.02^ab^	157.7 ± 13.29^a^
CP 4 days	3.95 ± 0.22^abcd^	163.85 ± 2.31^abcd^	38.87 ± 1.59^abc^	0.038 ± 0.003^abcd^	23.35 ± 4.19^abcd^
CP 4 days + Riva 5 mg	1.16 ± 0.13^abe^	79.133 ± 16.59^ae^	63.087 ± 6.03^be^	0.3 ± 0.03^abe^	153 ± 36.76^ae^
CP 4 days + Riva 7 mg	1.96 ± 0.12^abce^	127.71 ± 2.61^abce^	68.37 ± 2.86^e^	0.2 ± 0.019^abe^	128.11 ± 15.54^ae^


*Each value represents the mean of 6–8 experiments ± SEM. Statistical analysis was performed using one-way ANOVA followed by Tukey’s multiple comparisons test. ^*a*^Significantly different from control group value at *p* < 0.05; ^*b*^Significantly different from CP 1 day group value at *P* < 0.05; ^*c*^Significantly different from CP 2 day group value at *P* < 0.05; ^*d*^Significantly different from CP 3 day group value at *P* < 0.05; ^*e*^Significantly different from CP 4 day group value at *P* < 0.05.*

In addition, a significant elevation in GFR was observed after 1 day of CP injection followed by a gradual significant reduction in GFR after 2, 3 and 4 days as compared to normal control (**Table 1**). Levels of KIM-1 in the urine were markedly increased in days 1 and 4 from CP injection as compared to normal control with no changes in days 2 and 3 (**Figure 1C**). Pretreatment of animals with Riva in low dose (5 mg/kg b.w) and high dose (7 mg/kg b.w) significantly improved renal function tests. Riva in both doses significantly decrease Sr. Cr, BUN, urinary lipocaline-2 activity and urinary KIM-1 levels (**Table 1** and **Figures 1A–C**) compared to CP alone treated animals. In addition, it markedly increased urea/creat ratio, GFR and urinary GGT activity (**Table 1**) as compared to CP alone treated group.

### Effect of Cisplatin (CP) With or Without Rivaroxaban (Riva) on Hematological Parameters

In the current results, no significant change was observed in CBC counts in day 1 of CP injection with exceptional significant reduction in monocytes (Mon) count as compared to normal control. On the other hand, a significant reduction in WBCs and lymphocytes (Lymph) counts was observed starting from day 2 of CP administration. No changes were noticed in granulocytes (Gran) after 1, 2 and 3 days of CP injection (**Table 2**). After 4 days of single injection of CP, there were a significant increase in CBC counts including; WBCs, Lymph, Mon and Gran accompanied by a significant reduction in platelets count as compared to normal control (**Table 2**). Animal treated with CP with Riva at the two tested doses showed a significant reduction in CBC counts including; WBCs, Lymph, Mon and Gran with a significant increase in platelets count as compared to CP 4 days. Notably, these effects were not dose dependent.

**Table 2:** Effect of cisplatin (CP) with or without rivaroxaban (Riva) on hematological parameters (CBC and coagulation profile).

	Normal control	CP 1 day	CP 2 days	CP 3 days	CP 4 days	CP 4 days + Riva 5 mg	CP 4 days + Riva 7 mg
WBCs (10^3^/mm^3^)	8.31 ± 0.41	7.32 ± 0.78	4.89 ± 0.39^ab^	6.16 ± 0.62	13.07 ± 0.61^abcd^	9.7 ± 0.38^bcde^	10.26 ± 0.37^bcde^
Lymph (10^3^/mm^3^)	6.35 ± 0.46	5.88 ± 0.65	3.79 ± 0.31^ab^	4.67 ± 0.65	9.26 ± 0.32^abcd^	7.13 ± 0.42^cde^	6.93 ± 0.36^cde^
Mon (10^3^/mm^3^)	1.3 ± 0.09	0.64 ± 0.007^a^	0.66 ± 0.06^a^	1.03 ± 0.07	2.72 ± 0.31^abcd^	0.9 ± 0.05^e^	3.33 ± 0.15^abcde^
Gran (10^3^/mm^3^)	0.63 ± 0.08	0.65 ± 0.08	0.63 ± 0.14	0.87 ± 0.09	1.3 ± 0.10^abc^	0.73 ± 0.19^e^	0.49 ± 0.09^e^
Platelet (103/mm3)	586.13 ± 20.79	637 ± 14.53	705.2 ± 35.12^a^	736.4 ± 32.82^a^	408.38 ± 29.76^abcd^	661.6 ± 22.61^e^	662 ± 12.06^e^
Pt (sec)	10.10 ± 0.15	11.88 ± 0.33^a^	14.49 ± 0.36^ab^	14.17 ± 0.34^ab^	16.08 ± 0.47^abd^	12.63 ± 0.49^ace^	12.23 ± 0.32^acde^
PC (mg/dl)	121.24 ± 4.03	87.04 ± 4.23^a^	61.06 ± 2.51^ab^	73.03 ± 7.54^a^	54.04 ± 2.88^ab^	78.05 ± 5.09^ae^	78.6 ± 7.1^ae^
Ptt (sec)	19.63 ± 0.26	24.6 ± 0.69^a^	23.15 ± 1.16	24.83 ± 1.17^a^	24.38 ± 1.41^a^	26.17 ± 0.79^a^	28.83 ± 1.4^a^
INR	0.93 ± 0.01	1.07 ± 0.02^a^	1.25 ± 0.02^ab^	1.24 ± 0.01^ab^	1.4 ± 0.03^abcd^	1.1 ± 0.03^acde^	1.1 ± 0.04^acde^


*Each value represents the mean of 6–8 experiments ± SEM. Statistical analysis was performed using one-way ANOVA followed by Tukey’s multiple comparisons test. ^*a*^Significantly different from control group value at *p* < 0.05; ^*b*^Significantly different from CP 1 day group value at *P* < 0.05; ^*c*^Significantly different from CP 2 day group value at *P* < 0.05; ^*d*^Significantly different from CP 3 day group value at *P* < 0.05; ^*e*^Significantly different from CP 4 day group value at *P* < 0.05.*

Regarding to the coagulation profile markers, CP significantly increased Pt, ptt, and international normalized ratio (INR) starting from the 1st day with gradual significant increase from 2 days to 4 days, respectively, as compared to normal control (**Table 2**). Treatment with both doses of Riva significantly decreased Pt and INR with significant increase in Ptt as compared to CP 4 days (**Table 2**). Also, a significant reduction in prothrombin concentration (PC) was observed from the 1st day of CP injection as compared to normal control. Riva markedly increased PC concentration as compared to CP 4 days (**Table 2**).

### Effect of Cisplatin (CP) With or Without Rivaroxaban (Riva) on TF and Fibrin Expression

**FIGURE 2:**
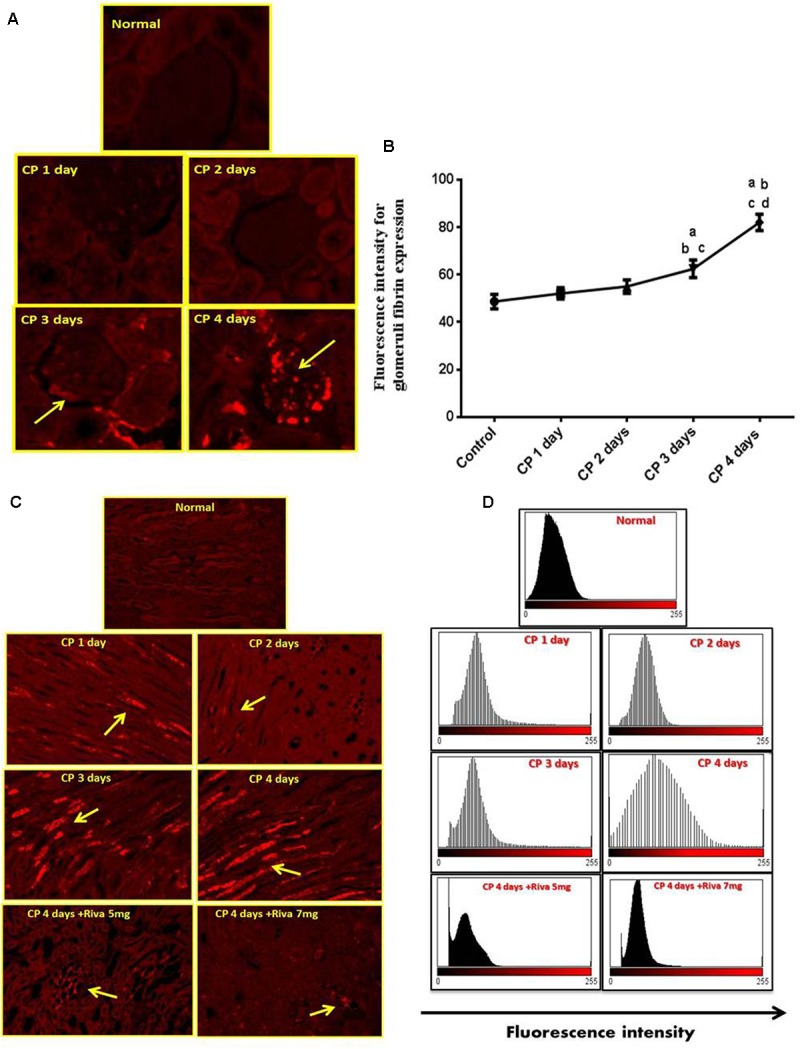
Effect of cisplatin (CP) with or without rivaroxaban (Riva) on fibrin expression: **(A)** Immunofluorescence staining of renal glomeruli of fibrin in normal and CP (6 mg/kg b.w) treated rats after 1, 2, 3 and 4 days, showing moderate to highly expression of fibrin in the glomerular cells after 3 and 4 days, respectively (yellow arrow) as compared to normal control. **(B)** Graphical presentation of fibrin fluorescence intensity in normal and cisplatin treated animals from day 1 to day 4 where (a) Significantly different from control group at *p* ≤ 0.01 (b) Significantly different from CP 1 day at *p* ≤ 0.01. (c) Significantly different from CP 2 day at *p* ≤ 0.01. (d) Significantly different from CP 3 day at *p* ≤ 0.01. **(C)** Immunofluorescence staining of renal tubular cells in normal and cisplatin (6 mg/kg) treated rats after 1, 2, 3, and 4 days in absence or presence of rivaroxaban, showing gradual increase in fibrin expression in renal tubular cells after 1 day till 4 days (yellow arrow). Basal expression of fibrin in the renal tubular cells of cisplatin pretreated with rivaroxaban. **(D)** Graphical histogram showing fluorescence intensity of fibrin in renal tubular cells of normal group and rats treated with cisplatin in absence or presence of rivaroxaban.

**FIGURE 3:**
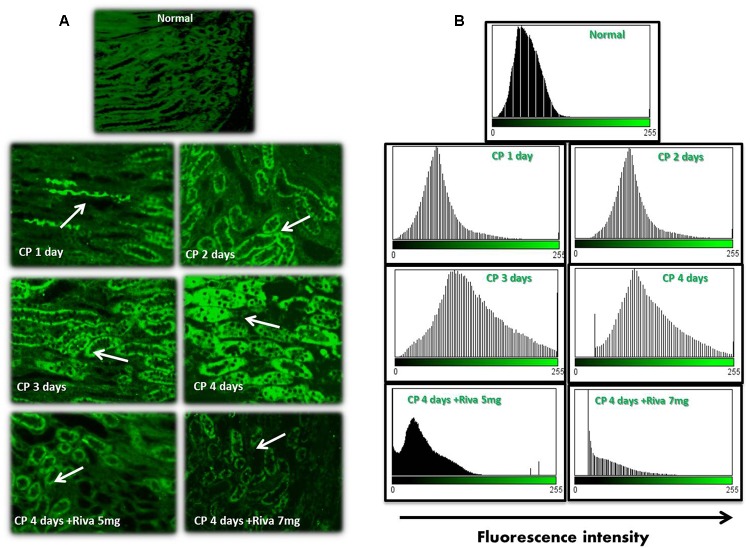
Effect of cisplatin (CP) with or without rivaroxaban (Riva) on tissue factor (TF) expression: **(A)** Immunofluorescence staining of renal tubular cells in normal rats and cisplatin (6 mg/kg) treated rats after 1, 2, 3 and 4 days in absence or presence of rivaroxaban. Gradual increase in TF expression in renal tubular cells staring from 1 day till 4 days which showed the highest expression of TF protein (white arrow). A significant low expression of TF in the renal tubular cells of cisplatin pretreated with rivaroxaban. **(B)** Histogram showing the fluorescence cells of normal group and rats treated with cisplatin in absence or presence of rivaroxaban.

Fibrin and TF proteins expressions were absent in renal glomeruli and tubular tissue of normal rat kidney tissues (**Figures 2A**, **3A**). In renal glomerular tissues, there was no expression of fibrin protein in rats injected with CP for 1 or 2 days while a significant increase in fibrin expression was observed after 3 and 4 days as compared to control group (**Figures 2A,B**). The highly expression of fibrin and TF proteins was observed in renal tubular cells after 1 day of CP administration with a gradual increase till 4 days (**Figures 2A–D**, **3A,B**) as compared to normal control. Treatment with Riva significantly decreased expression of fibrin and TF proteins in renal tubular cells as compared to CP 4 days (**Figures 2A–D**, **3A,B**).

### Effect of Cisplatin (CP) With or Without Rivaroxaban (Riva) on Renal Histological Features

**FIGURE 4:**
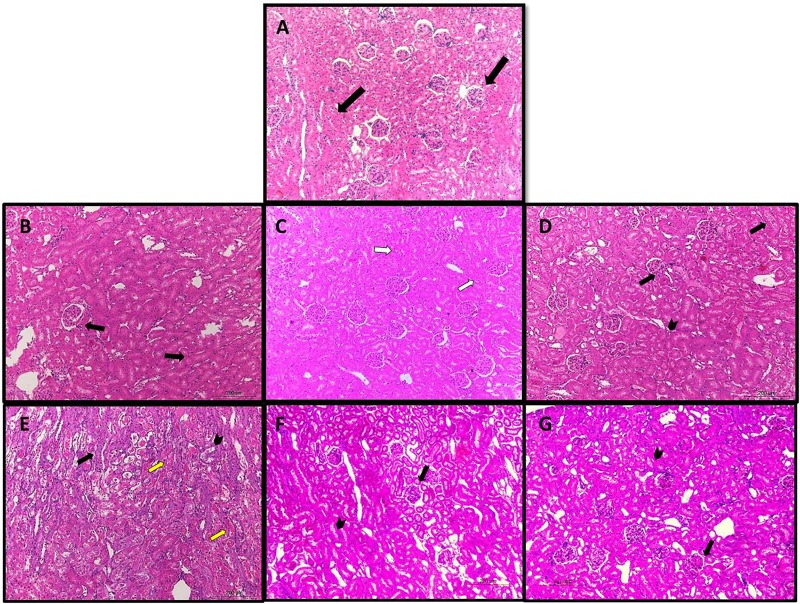
Photomicrograph of kidney sections of **(A)** normal rats showed normal histologic structure of glomeruli and renal tubules (black arrow). **(B)** Cisplatin treated rats after 1 day showed more or less normal histological structure of renal glomeruli and lining epithelium lining the renal tubules (black arrows). **(C)** 2 days after injection of cisplatin showed mild degenerative changes of renal epithelial degeneration associated with mild desquamation of tubular epithelial cells (white arrows). **(D)** Cisplatin treated rats after 3 days showed moderate epithelial degeneration of lining epithelium Additionally, glomeruli tufft are suffering from mild to moderate glomerulonephrosis (black arrow). focal leukocytic infiltration could be detected in the interstitial area (black head). **(E)** Cisplatin after 4 days showed severe degenerative changes and necrosis associated with desquamation of renal lining epitheliums (yellow arrow). Moreover glomeruli tuft are suffering from severe degenerative changes (black arrow). **(F)** Rats treated with cisplatin for 4 days with rivaroxaban 5 mg/kg showed moderate degenerative changes of renal epithelium (arrow head) associated with glomerulonephrosis (arrow). **(G)** Rats treated with cisplatin for 4 days with rivaroxaban 7 mg/kg showed moderate degenerative changes of renal epithelium (arrow head) associated with mild glomerulonephrosis (arrow). (HE X100).

Normal control animals showed normal histologic structure of glomeruli and renal tubules (**Figure 4A**). After 1 day of CP injection rats tissues showed more or less normal histological structure of renal glomeruli and lining epithelium lining the renal tubules (**Figure 4B**). In the 2nd day of CP administration mild degenerative changes of renal epithelial degeneration associated with mild desquamation of tubular epithelial cells were observed (**Figure 4C**). Cisplatin treated rats after 3 days showed moderate epithelial degeneration of lining epithelium. Additionally, glomeruli tufft showed mild to moderate glomerulonephrosis. Focal leukocytic infiltration could be detected in the interstitial area (**Figure 4D**).

Cisplatin after 4 days showed severe degenerative changes and necrosis associated with desquamation of renal lining epitheliums. Moreover glomeruli tuft showed severe degenerative changes (**Figure 4E**). Rats treated with CP for 4 days with Riva 5 and 7 mg/kg b.w showed moderate degenerative changes of renal epithelium associated with glomerulonephrosis (**Figures 4F,G**, respectively).

## Discussion

Activation of coagulation system is implicated in numerous pathological conditions including atherosclerosis (Day et al., 2008), Diabetes (El-Ghoroury et al., 2008), immune-mediated thrombosis and acute coronary syndromes or systemic inflammatory disease (George, 2008). To date, no previous study investigated the role of coagulation in the initiation of CP-induced ATN.

In the current study, CP caused renal impairment through affecting renal tubular cells causing tubular necrosis and cell death. This can be evidenced through the significant elevation of urinary KIM-1 and Lipocaline-2; renal specific proteins produced as a response of exposure of kidney tubules to toxins. In addition, CP significantly decreased urinary GGT activity. This tubular damage was reflected on the physiological function of kidney and this appeared in the significant increase in the Sr. Cr, cystatin-c and BUN concentration associated with significant reduction in GFR induced after 4 days of CP injection. These findings in line with the results obtained by Dirican et al. (2016) who reported that a single i.p. dose of CP 7.5 mg/kg to rats increased nephrotoxicity markers including serum cystatin-c and Lipocaline-2 as compared to normal control.

Tissue injury induced by CP injection may explain the marked changes in CBC and Pt. CBC including leukocytes, Lymph, and Mono were significantly decreased after 2 days and significantly increased 4 days after CP injection.

These can be explained depending on the fact that major leukocyte functions are completed in renal tissues therefore presence of leukocytes in the blood is a temporary state until crossing to the tissue (Goodman and Fuller, 2009). Leukocytes migration from blood to tissues cause leukopenia (Nourshargh and Alon, 2014) therefore, bone marrow may start to compensate this low level through increase leukocytes production as observed in our study by the 3 day of CP injection.

On the other hand, leukocytosis occurred after 4 days of CP injection. This may be as a reactive response of acute inflammation or tissue damage and degeneration (Agrawal and Verma, 2015). These results are in agreement with studies on blunt trauma patients who have shown higher WBC counts in the more severely injured patients (Santucci et al., 2008). In other study, it was reported that WBC count was significantly higher in patients with severe head injury compared to those with minor to moderate injury (Rovlias and Kotsou, 2001).

In addition, we observed a significant gradual increase in the Pt started 1 day after CP injection and peaked after 4 days. The increase in Pt was accompanied with significant decrease in the platelets count. This could be attributed to activation of platelets as a result of activation of coagulation cascade. Activated platelets liberates chemotactic substances and adhesion molecules which attract coagulation factors, inflammatory mediators, leukocytes and other platelets from blood to the injured tissue causing thrombocytopenia (Gresele et al., 2017). The hematological parameters are fit with the biochemical and histological results which confirmed by the renal impairment 4 days after CP injection.

One of the most important findings in this study is that CP increased expression of TF and fibrin proteins in renal tubular cells of rat’s kidney. In addition, we observed that the high expression of coagulation proteins was found earlier than the impairment of renal function which started 3 days after CP injection. Moreover, we noticed that TF and fibrin protein expressions were firstly elevated in the tubular cells followed by glomerular cells by the 4th day of CP injection.

These results are fit with the results reported by Ganey et al. (2007) who reported an increased expression of TF in acetaminophen-induced hepatotoxicity in mice. In addition, our results confirm the results obtained by Takabayashi et al. (2013) who reported that excessive fibrin deposition in the sub-mucosa of nasal polyps and its pathological role in patient with chronic rhino sinusitis.

The overexpression of fibrin and TF proteins in renal tubular cells may be due to the filtration of CP through the glomerular capillaries, taken up by kidney tubular cells, finally reaching its highest concentration in renal proximal tubules causing kidney injury and permanent decline in kidney function (Miller et al., 2010; Nasr and Saleh, 2014).

Cisplatin induced TF expression which is not expressed normally by cells (Key et al., 2007). TF acts as the key trigger of the coagulation cascade where it converts FX to FXa and consequently activates prothrombin (FII) to thrombin (FIIa) (Greer, 2004). Thrombin promotes clot formation by stimulating platelet activation and activating formation of fibrin, which polymerizes into actin fibers, and the platelets turns sticky and seals off the leakage. The formed clog of platelets is mechanically stabilized by the actin fibers (Guy et al., 2007). Excessive or uncontrolled clot production leads to thrombosis shutting off blood flow that supplies oxygen and nutrients to cells leading to cell death (Chambers and Scotton, 2012).

In the current study, Riva significantly decreased the expression of TF and fibrin in renal tubular cells which in turn keep normal structure and feature of the kidney. These were confirmed by histopathological, biochemical, hematological and other urinary biomarkers (as measured by western blot) which reflect the improvement of kidney tissue structure and function. Riva inhibits FXa which has a pivotal role in the coagulation system activation by converting pro-thrombin into thrombin (Rao and Pendurthi, 2005). Therefore, it plays an important role in inhibition of coagulation activation and in turn fibrin deposition, inflammation and tissue injury.

The effect of hemostasis on inflammation is supported by numerous reports that describe how the different components of coagulation system can regulate inflammation by exerting an influence on the endothelial cells, platelets and/or leukocytes (Daniel et al., 2008; Yoshida and Granger, 2009). Considering the cross-talk between coagulation and inflammation, it seems reasonable to hypothesize those anticoagulants as modulators of inflammation in general and those related to the coagulation system in particular. Gul Utku et al. (2015) reported the ability of rivaroxaban to be used in the treatment of colitis through anti-thrombotic and anti-inflammatory mechanisms.

## Conclusion

In conclusion, this study showed that blood hypercoagulability plays a crucial role in the pathogenesis of CP nephrotoxicity. In addition, using the anticoagulant, rivaroxaban, could contribute efficiently in protection against tubular damage induced by cisplatin.

## Author Contributions

ME performed the data collection, carried out the practical experiments and biochemical assay, performed the statistical analysis, and drafted the manuscript. BM participated in the design of the study, supervision of practical work, manuscript editing, and overall manuscript revision. MA-B participated in the design of the study and its co-ordination, and shared in the supervision of the practical study. AA-S conceived of the study, participated in its design, and performed overall revision on the study. AB carried out the western blotting study. All authors read and approved the final manuscript and contributed equally to this work.

## Conflict of Interest Statement

The authors declare that the research was conducted in the absence of any commercial or financial relationships that could be construed as a potential conflict of interest.

## Supplementary Material

Table S1 A pilot study of the effect of different doses of rivaroxaban on serum creatinine and BUN in CP treated rats.
