# Corrigendum: Interference With Coagulation Cascade as a Novel Approach to Counteract Cisplatin-Induced Acute Tubular Necrosis; an Experimental Study in Rats

**DOI:** 10.3389/fphar.2019.01259

**Published:** 2019-10-24

**Authors:** Mohamed G. Ewees, Basim A. S. Messiha, Ali A. Abo-Saif, Asmaa M. A. Bayoumi, Mohamed S. Abdel-Bakky

**Affiliations:** ^1^Department of Pharmacology and Toxicology, Faculty of Pharmacy, Nahda University, Beni-Suef, Egypt; ^2^Department of Pharmacology and Toxicology, Faculty of Pharmacy, Beni-Suef University, Beni-Suef, Egypt; ^3^Department of Pharmacology, Faculty of Medicine, Al-Azhar University, Cairo, Egypt; ^4^Department of Biochemistry, Faculty of Pharmacy, Minia University, Minia, Egypt; ^5^Department of Pharmacology and Toxicology, Faculty of Pharmacy, Al-Azhar University, Cairo, Egypt

**Keywords:** coagulation cascade, cisplatin, rivaroxaban, fibrin, tissue factor, nephrotoxicity

In the published article, there was an error in affiliation “1” and “2.” “Beni-Suef” was incorrectly written as “Beni Suef.” In affiliation 1, instead of “Department of Pharmacology and Toxicology, Faculty of Pharmacy, Nahda University, Beni Suef, Egypt,” it should be “Department of Pharmacology and Toxicology, Faculty of Pharmacy, Nahda University, Beni-Suef, Egypt.” In affiliation 2, instead of “Department of Pharmacology and Toxicology, Faculty of Pharmacy, Beni Suef University, Beni Suef, Egypt,” it should be “Department of Pharmacology and Toxicology, Faculty of Pharmacy, Beni Suef University, Beni-Suef, Egypt.”

The authors apologize for this error and state that this does not change the scientific conclusions of the article in any way. The original article has been updated.

