# Supplementary material for: Interference With Coagulation Cascade as a Novel Approach to Counteract Cisplatin-Induced Acute Tubular Necrosis; an Experimental Study in Rats
Source: Front Pharmacol. 2018 Oct 11;9:1155. doi: 10.3389/fphar.2018.01155 (PMC6193076; doi:10.3389/fphar.2018.01155)
Supplement: Table S1 — A pilot study of the effect of different doses of rivaroxaban on serum creatinine and BUN in CP treated rats. [file Table_1.DOCX]

|  | **Serum Cr.**  **(X ± S.E.)**  **(mg/dl)** | **BUN**  **(X ± S.E.)**  **(mg/dl)** |
| --- | --- | --- |
| **Normal control** | 0.52 ± 0.011 | 29.04 ± 1.7 |
| **CP 4 days** | 3.8 ± 0.04^a^ | 160.2 ± 4.51^a^ |
| **CP + Riva 1mg** | 3.1 ± 0.03^a^ | 153.25 ± 2.34^a^ |
| **CP + Riva 3mg** | 2.9 ± 0.10^a^ | 148.11 ± 7.10^a^ |
| **CP + Riva 5mg** | 1.2 ± 0.09^ab^ | 75.45 ± 2.31^ab^ |
| **CP + Riva 7mg** | 1.7 ± 0.08^ab^ | 110.12 ± 5.32^ab^ |

- Each value represents the mean of 6-8 experiments ± S.E.M.

- Statistical analysis was performed using one-way ANOVA followed by Tukey-Kramer multiple comparisons test where **(a)** significantly different from normal control value at p < 0.05, **(b)** significantly different from cisplatin 4 day value at p < 0.05.
